# Characterization of Post-Mortem pH Evolution and Rigor Mortis Process in Botucatu Rabbit Carcasses of Different Categories

**DOI:** 10.3390/ani14172502

**Published:** 2024-08-28

**Authors:** Daniel Rodrigues Dutra, Erick Alonso Villegas-Cayllahua, Giovanna Garcia Baptista, Lucas Emannuel Ferreira, Leandro Dalcin Castilha, Hirasilva Borba

**Affiliations:** 1 Department of Agricultural and Environmental Biotechnology, Faculty of Agriculture and Veterinary Sciences, São Paulo State University, Jaboticabal 14884-900, Brazil; 2 Department of Animal Science, State University of Maringá, Maringá 87020-900, Brazil

**Keywords:** carcass traits, meat quality, *Oryctolagus cuniculus*, pH curve, rabbit meat, slaughter liveweight

## Abstract

**Simple Summary:**

This study explored the effects of age, sex, and muscle type on post-mortem muscle acidification in Botucatu rabbits, addressing the gap between recommended meat quality assessment standards and industry practices. The World Rabbit Science Association (WRSA) suggests chilling carcasses for 24 h at 0–4 °C, while Brazilian practices often involve immediate freezing at temperatures below −18 °C. This study aimed to clarify how these practices affect muscle pH and rigor mortis development. Eighty Botucatu rabbits were categorized into young females and males, does, and bucks. Carcasses were chilled for 24 h at 4 °C. pH and temperature measurements were taken hourly for 24 h post-mortem in the *Longissimus lumborum* (LL) and Biceps femoris (BF) muscles. Rigor mortis was assessed manually alongside pH measurements. Botucatu does were heavier, with a less marked initial pH drop in the LL and delayed stabilization at 6 h post-mortem. Muscle acidification was more pronounced in the LL than in the BF. Rigor mortis set in at 5 h for young rabbits and bucks, and 6 h for does, resolving by 18 h. Thus, chilling rabbit carcasses for at least 18 h at 4 °C aligns WRSA guidelines with industry needs, ensuring effective rigor mortis and muscle-to-meat transformation.

**Abstract:**

The aim of the present study was to evaluate the characteristics of carcasses, monitor their pH evolution during the first 24 h post-mortem, and determine the time required for the establishment and resolution of rigor mortis in different categories of Botucatu rabbits. Live weight at slaughter, carcass weight, and yield were higher in 12-month-old animals compared to 3-month-old rabbits, regardless of sex. There was an effect of muscle type, age, and sex on the kinetics of muscle acidification, with the Biceps femoris showing a significantly higher pH than the Longissimus lumborum from 4 h post-mortem onward. The establishment of rigor mortis occurred at 5 h post-mortem in young rabbits and bucks and at 6 h post-mortem in does, along with pH stabilization, while the resolution of rigor occurred at 18 h post-mortem for all types of carcasses evaluated. In conclusion, Botucatu rabbit carcasses should be chilled continuously at 4 °C for a minimum of 18 h to ensure efficient rigor mortis progression and muscle-to-meat transformation throughout the carcass.

## 1. Introduction

The Commission of Harmonization of the World Rabbit Science Association (WRSA) has proposed standardizing several techniques used in registering carcass characteristics and other criteria related to the evaluation of rabbit meat quality (*Oryctolagus cuniculus domesticus*) [1]. Among these recommendations, it is suggested that the hot carcass be chilled for 24 h in a ventilated cold room at 0–4 °C to accurately record the drip loss percentage, dressing-out percentage, chilled carcass weight, and composition [1]. These measures differ from the practices routinely adopted in the rabbit meat industry, especially in Western developing countries such as Brazil, one of the largest producers of rabbit meat in Latin America, where hot carcasses are typically cooled immediately in fast-freezing tunnels at temperatures below −18 °C, usually in the pre-rigor stage. Freezing pre-rigor carcasses leads to significant changes in meat quality, affecting flavor, odor, color, and especially tenderness due to thaw rigor [2,3,4].

Still, early freezing of carcasses within the initial hours post-mortem is often justified as a cost-saving measure due to reduced operational times for chilling systems in cold chambers. In Brazil, for example, current legislation requires that carcasses be chilled or frozen before transportation for sanitary reasons [5,6], maintaining a maximum temperature of 4 °C [7], but there is no consensus on the required duration of cooling. Different cooling patterns can also influence the rate of muscle acidification and consequently affect the technological parameters of the meat [8], particularly the rate of pH drop in the various muscles, which is linked to the depletion of their energy reserves and the production of lactic acid in the early post-mortem hours [9].

Immediately after the animal’s death, muscle pH is near neutrality, dropping to a stable value (ultimate pH) within a few hours. Classical studies have shown that rabbit carcasses can achieve their final pH within the first 12 h post-mortem when stored at 0 °C [10]. Similarly, carcasses of New Zealand White females, slaughtered at 4–5 months of age with a live weight of 2.70 to 3.20 kg, can exhibit complete development and resolution of the rigor mortis process within 12 h following slaughter when chilled to 5 °C [11]. This evidence suggests that there may be a middle ground between the WRSA’s current technical recommendations and the actual needs of the industry.

Several factors influence muscle acidification during the early post-mortem stages, including pre-slaughter handling conditions [9], the time and temperature of carcass cooling [12], breed, age, sex, and the live weight of the animals at slaughter [13]. Paradoxically, few studies have tracked the behavior of muscle pH over the first 24 h post-mortem in commercially slaughtered rabbits [10,11], particularly as new genetic strains and breeds of different sizes have been introduced to the meat market, similar to what has been documented for other domestic species such as cattle [14,15], sheep [16], pigs [17], and even guinea pigs [18].

Therefore, to improve the accuracy of harmonization criteria and the standardization of research methods in meat rabbit farming, and to contribute to the production of higher-quality meat, this study aimed to evaluate carcass traits, characterize the rigor mortis process, and assess the effects of sex, age, and muscle type on the evolution of muscle pH during the first 24 h post-mortem in Botucatu rabbits under industrial conditions.

## 2. Materials and Methods

### 2.1. Animals

A total of 80 Botucatu rabbit carcasses were collected and distributed into four categories: females and young males at conventional slaughter age (90 days), and breeding males and disposal does (at 12 months of age) [*n* = 20/category]. The Botucatu genetic group is a double aptitude medium-sized lineage, selected for litter size and growth traits [19]. Originated from Norfolk 2000 hybrid rabbits (New Zealand White vs. California vs. Giant de Bouscat) [20], Botucatu rabbits have been successfully implemented in Brazil’s rabbit farms in recent years, also showing good results in performance, carcass characteristics, and meat quality [21]. These reasons are why they were chosen for this study.

The rabbits were raised under the same handling conditions in the Experimental Sector of Cuniculture of the Faculty of Agricultural and Veterinary Sciences of São Paulo State University (FCAV/UNESP), Jaboticabal Campus, Brazil (21°14′ S, 48°17′ W, 583 m altitude), and slaughtered in a commercial slaughterhouse for export rabbits, inspected by the Federal Inspection Service (S.I.F.). This study was approved by the Ethics Committee on the Use of Animals (CEUA) of the institution (protocol no. 1924/22).

The animals were weaned at 35 days of age and then individually housed in galvanized wire cages (flat-deck model) until they either reached slaughter age (90 days) or were culled at 12 months of age due to unsatisfactory reproductive performance (low libido, persistent anestrus, decrease in the number of kits per litter, and low maternal ability). Twenty rabbits from each group were randomly selected from the population raised at the experimental rabbit farm. Throughout that period, the rabbits received Coast-cross grass hay (*Cynodon dactylon* (L.) pers.) ad libitum and controlled single pelleted standard feed (“mixed feed”) (14% crude protein, 3% ethereal extract, 18% crude fiber, 15% mineral matter, 5% phosphorus, 10% calcium, and 13% moisture), according to each animal category [22].

The four groups were placed into conventional plastic cages (75 cm × 55 cm × 27 cm) and transported in the same refrigerated truck, set to an average temperature of 18 °C. The truck contained 10 fattening rabbits (413 cm^2^/animal) and 6 to 7 does and bucks (589–687 cm^2^/animal) loaded per cage, at densities of 24 rabbits/m^2^ and 15 to 17 rabbits/m^2^, respectively. Young females and males were mixed, while does were transported together, and bucks were kept aside by dividers to avoid aggression. All animals were individually identified on the right ear with a marker pen. The transport lasted 4 h, and the animals underwent 12 h of fasting and pre-slaughter rest in the slaughterhouse facilities [23]. After weighing the rabbits at the slaughterhouse, the bucks were kept separate from each other in the lairage area and were only placed with does. This was done to prevent aggression, ensuring that all categories remained homogeneously grouped until the time of slaughter.

### 2.2. Slaughter, Carcass Characteristics and pH

The animals were slaughtered according to the procedures routinely adopted by the slaughterhouse plant, without any interference in the slaughter flow, under all the operational requirements contained in the Regulation of Industrial and Sanitary Inspection of Products of Animal Origin (RIISPOA) [5]. Thus, the rabbits were stunned by electronarcosis, 110 V, 60 Hz, and 1.40 A for an average duration of 3 s, meeting the recommendations of Regulation (EC) N. 1099/2009 [24]. They were then hung upside down, exsanguinated by cutting the carotid artery and jugular vein, skinned, and finally eviscerated. The hot carcass weight (HCW) was recorded, and the hot carcass yield (HCY) calculation was [adapted and] performed according to Blasco and Houhayoun [1]:

$$HCY=\frac{Hot\;carcass\;weight}{Live\;weight\;at\;slaughter}\times 100$$

The following parts were not considered hot carcasses as these were removed along the slaughter line: the head, the liver, the kidneys, and the organs of the chest and neck. The culture of marketing rabbit carcasses with heads is absent in Brazil [5], so it was not included in the calculation of carcass characteristics. Then, the hot carcasses were randomly stored in a ventilated cold chamber set to a temperature of 4 °C for 24 h, with the air circulation speed adjusted (0.5 to 3.0 m/s) to ensure proper chilling. This temperature was set based on the maximum chilled storage temperature recommended by the current Brazilian Normative Instruction for beef, pork, and chicken [7], as rabbit meat is not covered by Brazilian legislation, and it aligns with the standards adopted by the unit.

The pH of the 80 carcasses was measured in situ using a Testo digital pH meter (model 205) with a penetration electrode, treating each carcass as an individual experimental unit. Measurements were taken on the left side of each carcass, inserting the electrode approximately 5 cm into the *Longissimus lumborum* (LL) muscle at the height of the 5th lumbar vertebra and into the *Biceps femoris* (BF) muscle through the inner thigh [25]. The measurements were taken every 1 h in all carcasses until 24 h post-mortem were completed. The first measurement was performed on the hot carcass shortly after evisceration. The pH meter was equipped with automatic temperature compensation to ensure accurate measurements. All animals were slaughtered on the same day and stored in the same cold chamber. After 24 h post-mortem, the carcasses were weighed again to obtain the weight of the cold carcass (reference carcass weight) and the yield of the cold carcass (dressing out percentage, DoP), according to Blasco and Houhayoun [1]:

$$DoP=\frac{Cold\;carcass\;weight}{Live\;weight\;at\;slaughter}\times 100$$

### 2.3. Rigor Mortis Evaluation

The development of rigor mortis was also manually evaluated, concomitantly with pH measurements, in a similar manner to that performed by Sánchez-Macías et al. [18] for guinea pig carcasses, adapted from Varetto and Curto [26]. The left thigh was suspended with one hand and the left femorotibiopatelar joint with the other so the knee could be flexed. At first, when it was no longer possible to flex the knee joint, or even flex it a few degrees, the establishment of rigor mortis was considered. In a second moment, when the knee could be flexed again, the resolution of rigor mortis was considered.

### 2.4. Statistical Analysis

Statistical analyses were performed using the SAS statistical software (version 9.1, SAS Institute Inc., Cary, NC, USA). The “General Linear Models” procedure (Proc GLM) was used to evaluate the carcass characteristics, considering the effects of sex and age at slaughter, and to characterize the pH evolution in the first 24 h post-mortem, accounting for the effects of age, sex, and muscle at each time point studied. Proc GLM for repeated time measures was further used to evaluate the effect of post-mortem time on pH in distinct analyses for each age, sex, and muscle. The Tukey test was used to establish statistical differences between the means, with significance set at *p* < 0.05. Fischer’s exact test was also used to determine the frequency of occurrence of femorotibiopatelar joint flexion, also with significance set at *p* < 0.05.

## 3. Results and Discussion

### 3.1. Live Weight at Slaughter, Weight, and Yield of Hot and Cold Carcasses

Significant differences were observed in live weight at slaughter (LW), with older rabbits weighing more than younger ones. At 12 months of age, the females weighed 4.63 kg, compared to 3.04 kg at 3 months of age. For the males, the weight was 3.76 kg at 12 months and 2.85 kg at 3 months. This increase in live weight aligns with observations by Machado et al. [27], who reported minimum weights of 4.20 kg for both pure Botucatu does and Botucatu × White New Zealand cross-bred females at 156 days of age. Similarly, Moura and Fernandes [28] noted comparable weights of 4.60 kg for Botucatu does at 34 weeks. A significant interaction (*p* = 0.002) was observed between sex and age for LW (Table 1). While no differences in live weight were observed among younger rabbits, significant differences emerged in adulthood, with females outweighing males. At conventional slaughter ages of 75 to 90 days, rabbits have not yet reached sexual maturity. Sexual dimorphism in domestic rabbits typically manifests after the 15th week [29], with females generally exhibiting superior weight gain due to greater amounts of adipose tissue and larger gastrointestinal tracts [21,30,31]. This pattern explains the findings for the Botucatu lineage in this study, where the does were 18.85% heavier than the bucks.

This study also found a significant interaction between sex and age for hot carcass weight (HCW) and cold carcass weight (CCW) (*p* = 0.001 and *p* = 0.001, respectively) (Table 1). Older rabbits exhibited higher HCW and CCW, reflecting their greater body mass compared to the younger rabbits. This finding aligns with Bianospino et al. [21], who observed that Botucatu rabbits at 91 days of age produced cold carcasses weighing 1.50 kg from 2.93 kg of live weight. Machado et al. [27] and Moura and Fernandes [28] similarly reported comparable weights, further supporting the impact of age on carcass weight. This aligns with Dalle Zotte et al. [32], who reported heavier carcasses in older animals and no sex differences in young rabbits. The literature in general indicates that sex has minimal or no effect on carcass traits in young rabbits at the conventional slaughter ages of 75 to 90 days [33,34], which is consistent with the findings for 3-month-old rabbits in the present study. Hernández et al. [35] found that older rabbits selected for growth had higher live weights and carcass yields due to a lower organ percentage and higher loin percentage. Deltoro and López [30] also reported that in New Zealand White and California breeds, carcass yield and meat-to-bone ratio increased with age. 

In intensive systems, short birth intervals lead to the production of 50 to 60 kits per year and a productive lifespan of up to 12 months for does, as observed in technified Brazilian rabbit farms [36]. This system can result in high turnover rates, with annual replacement rates approaching 120%, as already seen in countries with traditional rabbit meat production, such as Spain [37]. In southern countries like Brazil, the local market demands heavier rabbit carcasses to meet the need for larger cuts of meat [38], underscoring the potential of culling rabbits in the final stages of reproduction for consumption. However, there is a notable lack of information on carcass traits and meat quality for this category, highlighting the relevance of this study to the rabbit meat industry.

Significant differences in hot carcass yield (HCY) and cold carcass yield (CCY) were noted, with older rabbits showing higher yields. Specifically, HCY increased from 51.74% at 3 months to 53.78% at 12 months, and CCY rose from 50.05% to 52.36% over the same period (*p* = 0.005 and *p* = 0.002, respectively). This increase is attributed to ongoing muscular development and allometric fat accumulation with age, leading to heavier carcasses and improved yields [33]. In the first study evaluating the carcass characteristics and meat quality of the Botucatu lineage, Bianospino et al. [21] observed increased carcass yields with age. Pure rabbits exhibited a reference carcass yield of 53.80% at 42 days, which progressively increased to 62.10% by 91 days. This increase is directly related to the growth in weight of both forelimbs and hindlimbs, particularly due to significant muscle deposition in the hindquarter. It is important to highlight that in our study, carcasses did not include the head or viscera, unlike in the last cited study where these components were considered part of the carcass. Zeferino et al. [39] reported the yields of 54.89% of pure Botucatu rabbits and 55.24% of Botucatu × New Zealand White crossbreds at 70 days of age. Several studies using other breeds and genetic lineages have also found that the carcass yield of rabbits can increase depending on age [40,41]. These results align with our findings and confirm the suitability of the Botucatu line for meat production. In addition, as rabbits grow, the allometric coefficient of their organs typically decreases, while the meat-to-bone ratio increases. This shift leads to an enhancement in carcass yield with increasing age at slaughter [42,43].

### 3.2. Evolution of pH

The mean pH values recorded in the *Longissimus lumborum* (LL) and *Biceps femoris* (BF) muscles during the first 24 h post-mortem in Botucatu rabbits are presented in Table 2, where the effect of post-mortem time was considered as a function of the animal’s sex and age for each muscle evaluated. Significant differences were observed in muscle pH over the analyzed period for both the LL and BF muscles. The pH values decreased as the post-mortem time increased (*p* < 0.05) in the LL and BF of male and female rabbits, regardless of age. 

For the LL, pH values were higher (*p* < 0.05) between 0 and 2 h post-mortem in females compared to other times, regardless of age. The LL of males, however, showed the highest pH value (*p* < 0.05) up to 1 h post-mortem, as seen in the BF of both ages and sexes. The decline in pH is attributed to the consumption of glycolytic reserves after slaughter, leading to lactic acid and H^+^ ion accumulation from glycogen breakdown, the primary muscle energy source [44,45]. Typically, muscle pH decreases from near neutral to a stable value between 5.3 and 6.0 post-mortem [13], consistent with the findings of this study. 

At 0 h post-mortem, the pH ranged from 6.52 to 6.74 in the LL and from 6.74 to 6.80 in the BF. The pH dropped sharply and stabilized around 2 h post-mortem in the LL of bucks and 5 h post-mortem in the LL of young rabbits. Interestingly, the pH in the LL of does began its stabilization process later, starting at 6 h post-mortem.

The pH of the BF also stabilized later for young females, around 7 h post-mortem, whereas the pH values for young males, bucks, and does stabilized earlier, between 2 and 4 h post-mortem. Notably, these pH values were higher compared to those observed in the LL.

Ikeuchi et al. [10] found that the pH of the *Longissimus thoracis et lumborum* (LTL) dropped from 7.1 to 6.5 at 2 h post-mortem at 37 °C, stabilizing around 5.6 within the first 4 h. The same authors found that at 0 °C, pH decreased more gradually, reaching 5.9 only at 12 h post-mortem, corroborating findings by Bendall [44] on the significant impact of carcass cooling temperature on pH behavior. However, Ikeuchi et al. [10] did not report animal weight, sex, breed, or age, nor did they follow commercial slaughterhouse parameters.

The differences in muscle acidification rates relate to muscle fiber characteristics and glycolytic potential [45]. Muscle fibers are categorized into types I (slow oxidative), II (fast oxidative), and IIB (fast glycolytic), with recent proposals suggesting four skeletal muscle isoforms: I, IIa, IIx, and IIb [46,47]. The proportion of these fibers varies by breed and muscle [48,49], with high glycolytic metabolism leading to rapid pH decline and distinct muscle fiber acidification kinetics [49,50,51].

The pH stability with higher values in the BF is due to its slower contraction, higher oxidative metabolism, and lower glycolytic potential, making it less acidic than the LL [13,52]. Dalle Zotte et al. [53] confirmed higher oxidative activity and pH in rear limb muscles compared to the LL in growing rabbits. Does, being heavier and having more glycolytic fibers in the LL, exhibited delayed pH stabilization compared to the BF, also aligning with Dalle Zotte et al. [54], who found glycolytic metabolic activity in the LL ceased later than in the BF.

**Figure 1:**
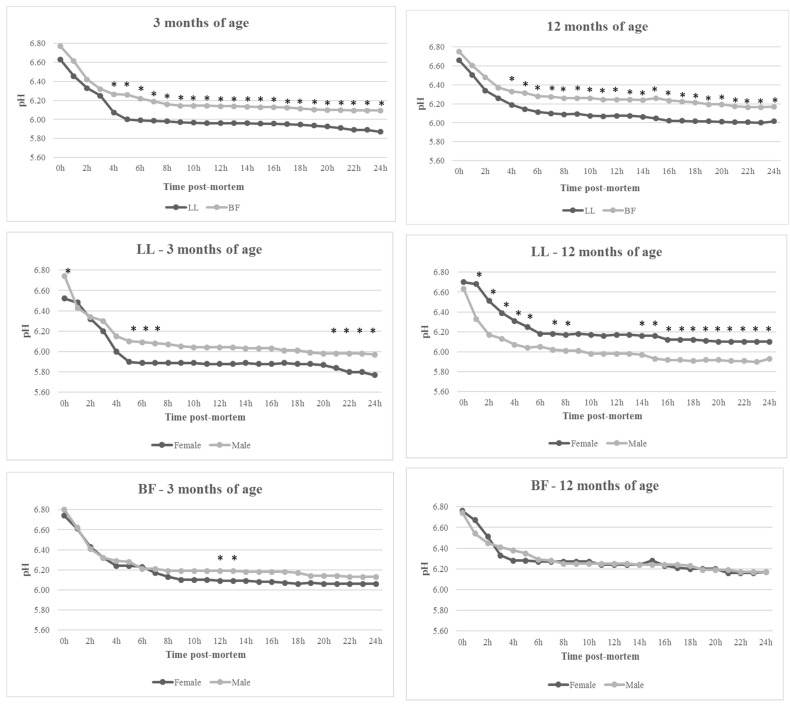

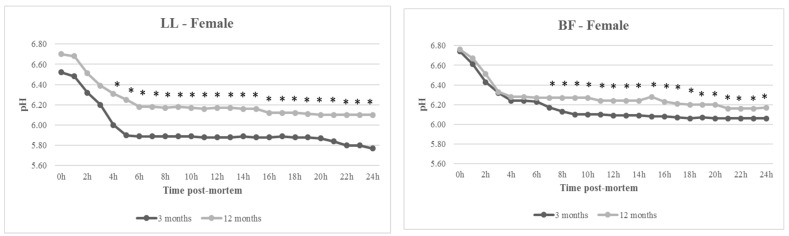
Evolution of pH of *Longissimus lumborum* (LL) and *Biceps femoris* (BF) in female and male Botucatu rabbits slaughtered at 3 and 12 months of age during first 24 h post-mortem. Values within time with * differ significantly at *p* < 0.05.

From 4 h post-mortem, muscle type significantly affected pH, with the BF showing higher values at each time analyzed until 24 h, regardless of age (Figure 1). This demonstrates less efficient acidification of the BF compared to the LL, attributed to its glycolytic fiber composition [54,55]. Tůmová et al. [55] reported over 90% white, glycolytic fibers in Czech rabbits’ LTL, while Dalle Zotte and Ouhayoun [54] observed 98% white glycolytic fibers in the LL.

In young animals, mean pH values were 6.27 (BF) and 6.08 (LL) at 4 h post-mortem, decreasing to 6.10 and 5.87, respectively, by 24 h, showing a 0.23-unit difference between muscles, which is low compared to the 0.70-unit difference reported in the literature [47]. In older animals, pH values were 6.33 (BF) and 6.19 (LL) at 4 h post-mortem, decreasing to 6.17 and 6.02, respectively, at 24 h, with a 0.15-unit difference. These findings are consistent with the literature [56,57,58,59], particularly regarding higher pH values in the BF due to its oxidative nature in comparison to the LL, as illustrated in Figure 1.

The effect of sex and age on muscle pH during the first 24 h post-slaughter was observed. In young rabbits, the initial pH of the LL was higher in males (6.74) compared to females (6.52). The published literature on the effect of sex on muscle pH in production rabbits is extensive but inconclusive, with minimal effects observed in young animals [53,56,60,61]. However, elevated pH values in the LTL of young males compared to females have been reported [57,62,63]. Barrón et al. [64] also observed a sex-related influence on the pH_20min_ of the LTL in rabbits at 70 days of age, with higher values in males. This aligns with our findings and could be related to the physiological stress associated with agonistic behavior among pubescent males, which affects muscle carbohydrate levels and elevates initial pH. By 90 days of age, male rabbits frequently exhibit agonistic behavior as they establish social hierarchies [65,66,67]. When mixed into a new group of young rabbits at loading up to slaughter, this stress likely reduces muscle carbohydrate reserves, increases liver carbohydrate levels, and contributes to the higher pH observed in males.

This effect was not observed in adult males, as the bucks were not mixed with other males in the lairage area but only with does, to avoid aversive behaviors. This likely minimized chronic stress in these animals and consequently allowed for a gradual decrease in pH during the first 24 h post-mortem. Studies on culled rabbits are still lacking, highlighting the need for further research on the physicochemical properties of their meat at various stages of production. Moreover, male rabbits tend to have a higher proportion of aerobic fibers with lower muscle glycogen content, which may have directly influenced the more pronounced pH decrease later observed in the LTL of young males [32,46]. Stress-induced catecholamine release can accelerate glycolysis, ATP hydrolysis, and muscle acidification [13], as indicated by the rapid pH drop in the first hour post-mortem.

In 12-month-old rabbits, the pH in the LL was lower (*p* < 0.05) for bucks compared to does, decreasing from 6.33 to 5.93 in males and from 6.68 to 6.10 in females from 1 to 24 h post-mortem. The lighter weight of bucks resulted in efficient acidification proportional to carcass weight, challenging the idea that heavier rabbits always have more acidic meat [13,68]. The higher initial pH in does compared to young females may indicate insufficient lactic acid production and slower ATP depletion, suggesting a need for further biochemical studies. In males, age had no effect on muscle pH, with similar values over the 24 h post-mortem in the LL (3 months: 6.74_pH0_ → 5.97_pHu_; 12 months: 6.63_pH0_ → 5.93_pHu_) and the BF (3 months: 6.80_pH0_ → 6.13_pHu_; 12 months: 6.74_pH0_ → 6.17_pHu_). This consistency reflects similar muscle acidification rates across ages in Botucatu males. Bini et al. [69] and Battaglini et al. [70] also observed no pH decrease with rabbit growth in five different muscles, reinforcing these findings.

### 3.3. Establishment and Resolution of Rigor Mortis

The relative frequency (%) of Botucatu rabbits that presented femorotibiopatelar flexion during the first 24 h post-mortem is presented in Table 3, where the effect of post-mortem time was considered for each evaluated category. The highest frequency (*p* < 0.05) of femorotibiopatelar flexion in carcasses of 12-month-old females was observed between 0 h and 5 h and between 18 h and 24 h post-mortem. In contrast, carcasses from other categories showed a higher frequency of flexion (*p* < 0.05) before 5 h and after 17 h post-mortem.

These observations indicate that rigor mortis was established by 5 h post-mortem for all categories, except for does, where rigor mortis began at 6 h post-mortem. This timing aligns with the stabilization of muscle pH, consistent with Bendall’s rigor mortis standards for rabbits [44]. Bendall observed that rigor mortis initiates when 1/3 to 1/5 of muscle fiber ATP is depleted, resulting in reduced sarcomere length and increased resistance to stretching due to the formation of the actomyosin complex. The author also demonstrated that, in carcasses stored at 38 °C, psoas muscle shortening began approximately one hour before the onset of resistance to stretching at 4 h post-mortem, with a reduction of up to 25% of its initial length. In the present study, this timeframe may have been extended by 1 to 2 h due to the lower chilling temperature of the carcasses (4 °C), which positively delayed the biochemical processes associated with the progression of rigor mortis.

Once established, rigor mortis persisted for about 10–11 h without variation among animal categories. At approximately 18 h post-slaughter, rigor mortis began to resolve, indicated by the return of femorotibiopatelar joint flexibility. This marks the onset of the post-rigor phase, where muscle extensibility gradually increases due to proteolytic degradation by calpains and calpastatins, restoring some degree of extensibility [71].

Generally, muscle pH decreases until rigor mortis is established, after which it stabilizes, as observed in other domestic species [14,15,16,17,18,72]. Sánchez-Macías et al. [18] reported similar results, with rigor mortis beginning between 4 h 50 min and 5 h 20 min post-mortem for guinea pigs, persisting for about 10 h before resolution started between 12 and 15 h post-mortem.

Based on these findings, it is crucial to avoid processes that could disrupt the early cold chain and affect rigor mortis development, such as processing, freezing, packaging, and early cooking, until 18 h of chilling at a constant 4 °C. Failure to adhere to this could compromise muscle-to-meat conversion and the final product’s microbiological and physicochemical quality, impacting attributes like tenderness, color, and flavor [8,73]. Geesink et al. [74] noted similar issues in lambs, with severe muscle contractions and increased hardness in meat cooked in the pre-rigor state compared to post-rigor samples. 

Preventing thaw rigor losses is essential, as this phenomenon occurs when carcasses or cuts are frozen before rigor mortis is fully established. Thaw rigor results in irreversible tenderness changes due to severe sarcomere shortening and exudate release caused by the sudden release of Ca^+2^ ions in the sarcoplasm [71]. Rabbit carcasses are particularly sensitive to thaw rigor, with varying responses depending on the muscle. For example, the psoas major muscle, while resistant to cold shortening, can experience a 73% reduction in sarcomere length due to thaw rigor, whereas the Semitendinosus muscle shows about 39% shortening [75].

Thus, this study provides valuable insights into rigor mortis in Botucatu rabbits and offers a reference for future research aimed at understanding the physicochemical characteristics of cuts from this promising breed, currently being adapted in Brazilian rabbit farms.

## 4. Conclusions

Botucatu does are heavier than bucks, resulting in greater carcass yield, and both weight and yield increase with age. These differences in weight sex, and muscle type affect the efficiency of post-mortem muscle acidification. The initial acidification of the *Longissimus lumborum* in females is less pronounced than in males, leading to delayed stabilization in does at 6 h. The *Biceps femoris* shows early acidification by 1–2 h post-mortem, with young females stabilizing later at 7 h. Notably, acidification kinetics in the *Longissimus lumborum* become evident from 4 h post-mortem. Rigor mortis sets in at 5 h post-mortem for young rabbits and bucks, and at 6 h for does, resolving by 18 h.

Thus, the continuous chilling of rabbit carcasses for a minimum of 18 h at 4 °C represents the practical intersection point between the WRSA’s technical guidelines and industry requirements. This approach ensures effective rigor mortis progression and muscle-to-meat transformation throughout the carcass, regardless of weight, sex, or age, while preventing quality losses associated with freezing during the pre-rigor stage.

## Figures and Tables

**Table 1:** Interaction between age and sex for LW, HCW, and CCW (range) and means for HCY, and CCY in Botucatu rabbits by age and sex. *p*-values are presented.

Sex (*n* = 20)	Age (*n* = 20)		*p*-Value			CV (%)
	3 Months	12 Months				
	*LW* (kg)		*p* (A)	*p* (S)	*p* Int. (AxS)	
Female	3.04 (2.70–3.50) ^Ab^	4.63 (4.20–5.40) ^Aa^	<0.001	<0.001	0.002	8.97
Male	2.85 (2.40–3.10) ^Ab^	3.76 (3.30–3.90) ^Ba^				
	*HCW* (kg)		*p* (A)	*p* (S)	*p* Int. (AxS)	
Female	1.55 (1.40–1.80) ^Ab^	2.49 (2.25–3.08) ^Aa^	<0.001	<0.001	0.001	9.07
Male	1.49 (1.36–1.64) ^Ab^	2.01 (1.84–2.32) ^Ba^				
	*CCW* (kg)		*p* (A)	*p* (S)	*p* Int. (AxS)	
Female	1.51 (1.37–1.75) ^Ab^	2.44 (2.20–3.01) ^Aa^	<0.001	<0.001	0.001	9.05
Male	1.44 (1.30–1.57) ^Ab^	1.95 (1.79–2.27) ^Ba^				
			HCY (%)			CCY (%)
Age (A)						
3 months			51.74 ^b^			50.05 ^b^
12 months			53.78 ^a^			52.36 ^a^
Sex (S)						
Female			52.58 ^a^			51.20 ^a^
Male			52.96 ^a^			51.21 ^a^
*p*-value						
*p* (A)			0.005			0.002
*p* (S)			0.472			0.845
*p* Int. (A × S)			0.231			0.223
CV (%)			4.02			4.09

Means followed by different letters in columns (uppercase ^A,B^) and rows (lowercase ^a,b^) differ significantly according to the Tukey test (*p* < 0.05). HCY: hot carcass yield; CCY: cold carcass yield; LW: live weight at slaughter; HCW: hot carcass weight; CCW: cold carcass weight; A: age; S: sex; Int: interaction; CV: coefficient of variation.

**Table 2:** Evolution of pH (± SD) during the first 24 h post-mortem in muscles *Longissimus lumborum* and *Biceps femoris* of male and female Botucatu rabbits slaughtered at different ages. *p*-values are presented.

Time Post-Mortem	pH							
	*Longissimus lumborum*				*Biceps femoris*			
	3 Months Botucatu Rabbits (*n* = 20)		12 Months Botucatu Rabbits (*n* = 20)		3 Months Botucatu Rabbits (*n* = 20)		12 Months Botucatu Rabbits (*n* = 20)	
	Female	Male	Female	Male	Female	Male	Female	Male
0 h	6.52 ± 0.06 ^A^	6.74 ± 0.07 ^A^	6.70 ± 0.07 ^A^	6.63 ± 0.05 ^A^	6.74 ± 0.05 ^A^	6.80 ± 0.07 ^A^	6.76 ± 0.07 ^A^	6.74 ± 0.10 ^A^
1 h	6.48 ± 0.06 ^A^	6.43 ± 0.07 ^B^	6.68 ± 0.08 ^A^	6.33 ± 0.04 ^AB^	6.61 ± 0.06 ^A^	6.62 ± 0.09 ^AB^	6.67 ± 0.05 ^A^	6.54 ± 0.05 ^AB^
2 h	6.32 ± 0.07 ^ABC^	6.34 ± 0.07 ^BC^	6.51 ± 0.06 ^AB^	6.17 ± 0.07 ^BC^	6.43 ± 0.05 ^B^	6.41 ± 0.08 ^BC^	6.51 ± 0.06 ^B^	6.45 ± 0.05 ^BC^
3 h	6.20 ± 0.07 ^BC^	6.3 ± 0.07 ^BCD^	6.39 ± 0.05 ^BC^	6.13 ± 0.07 ^BC^	6.32 ± 0.03 ^BC^	6.32 ± 0.07 ^BC^	6.33 ± 0.02 ^C^	6.41 ± 0.06 ^BC^
4 h	6.00 ± 0.09 ^CD^	6.15 ± 0.09 ^BCD^	6.31 ± 0.03 ^BC^	6.07 ± 0.05 ^BC^	6.24 ± 0.02 ^CD^	6.29 ± 0.13 ^C^	6.28 ± 0.03 ^CD^	6.38 ± 0.05 ^BCD^
5 h	5.90 ± 0.07 ^DE^	6.10 ± 0.06 ^CDE^	6.25 ± 0.04 ^BC^	6.04 ± 0.03 ^C^	6.24 ± 0.01 ^CD^	6.28 ± 0.05 ^C^	6.28 ± 0.03 ^CD^	6.35 ± 0.06 ^BCD^
6 h	5.89 ± 0.06 ^DE^	6.09 ± 0.04 ^CDE^	6.18 ± 0.01 ^CD^	6.05 ± 0.04 ^C^	6.23 ± 0.01 ^CDE^	6.21 ± 0.04 ^C^	6.27 ± 0.01 ^CD^	6.29 ± 0.02 ^BCD^
7 h	5.89 ± 0.03 ^DE^	6.08 ± 0.05 ^CDE^	6.18 ± 0.01 ^CD^	6.02 ± 0.05 ^C^	6.17 ± 0.01 ^DEFG^	6.21 ± 0.05 ^C^	6.27 ± 0.02 ^CD^	6.28 ± 0.03 ^CD^
8 h	5.89 ± 0.04 ^DE^	6.07 ± 0.05 ^CDE^	6.17 ± 0.01 ^CD^	6.01 ± 0.05 ^C^	6.13 ± 0.01 ^DEFG^	6.19 ± 0.04 ^C^	6.27 ± 0.01 ^CD^	6.25 ± 0.03 ^CD^
9 h	5.89 ± 0.02 ^DE^	6.05 ± 0.05 ^CDE^	6.18 ± 0.08 ^CD^	6.01 ± 0.05 ^C^	6.10 ± 0.01 ^FG^	6.19 ± 0.04 ^C^	6.27 ± 0.01 ^CD^	6.25 ± 0.08 ^CD^
10 h	5.89 ± 0.02 ^DE^	6.04 ± 0.05 ^DE^	6.17 ± 0.07 ^CD^	5.98 ± 0.05 ^C^	6.10 ± 0.01 ^FG^	6.19 ± 0.04 ^C^	6.27 ± 0.01 ^CD^	6.25 ± 0.03 ^CD^
11 h	5.88 ± 0.01 ^DE^	6.04 ± 0.06 ^DE^	6.16 ± 0.06 ^CD^	5.98 ± 0.05 ^C^	6.09 ± 0.01 ^G^	6.19 ± 0.08 ^C^	6.24 ± 0.02 ^CD^	6.25 ± 0.03 ^CD^
12 h	5.88 ± 0.01 ^DE^	6.04 ± 0.06 ^DE^	6.17 ± 0.05 ^CD^	5.98 ± 0.06 ^C^	6.09 ± 0.02 ^G^	6.19 ± 0.05 ^C^	6.24 ± 0.01 ^CD^	6.25 ± 0.01 ^CD^
13 h	5.88 ± 0.02 ^DE^	6.04 ± 0.06 ^DE^	6.17 ± 0.05 ^CD^	5.98 ± 0.08 ^C^	6.09 ± 0.01 ^G^	6.19 ± 0.06 ^C^	6.24 ± 0.01 ^CD^	6.25 ± 0.01 ^CD^
14 h	5.89 ± 0.01 ^DE^	6.03 ± 0.04 ^DE^	6.16 ± 0.05 ^D^	5.97 ± 0.08 ^C^	6.09 ± 0.01 ^G^	6.18 ± 0.05 ^C^	6.24 ± 0.01 ^CD^	6.24 ± 0.06 ^CD^
15 h	5.88 ± 0.02 ^DE^	6.03 ± 0.05 ^DE^	6.16 ± 0.04 ^D^	5.93 ± 0.07 ^C^	6.08 ± 0.02 ^G^	6.18 ± 0.07 ^C^	6.28 ± 0.01 ^CD^	6.24 ± 0.05 ^CD^
16 h	5.88 ± 0.02 ^DE^	6.03 ± 0.05 ^DE^	6.12 ± 0.04 ^D^	5.92 ± 0.07 ^C^	6.08 ± 0.01 ^G^	6.18 ± 0.06 ^C^	6.23 ± 0.01 ^CD^	6.24 ± 0.09 ^CD^
17 h	5.89 ± 0.01 ^DE^	6.01 ± 0.05 ^DE^	6.12 ± 0.03 ^D^	5.92 ± 0.08 ^C^	6.07 ± 0.01 ^G^	6.18 ± 0.05 ^C^	6.21 ± 0.02 ^CD^	6.24 ± 0.02 ^CD^
18 h	5.88 ± 0.01 ^DE^	6.01 ± 0.03 ^DE^	6.12 ± 0.04 ^D^	5.91 ± 0.07 ^C^	6.06 ± 0.02 ^G^	6.17 ± 0.05 ^C^	6.20 ± 0.02 ^CD^	6.23 ± 0.03 ^CD^
19 h	5.88 ± 0.01 ^DE^	5.99 ± 0.05 ^E^	6.11 ± 0.04 ^D^	5.92 ± 0.06 ^C^	6.07 ± 0.01 ^G^	6.14 ± 0.05 ^C^	6.20 ± 0.01 ^CD^	6.19 ± 0.05 ^D^
20 h	5.87 ± 0.02 ^DE^	5.98 ± 0.05 ^E^	6.10 ± 0.02 ^D^	5.92 ± 0.05 ^C^	6.06 ± 0.01 ^G^	6.14 ± 0.03 ^C^	6.20 ± 0.01 ^CD^	6.19 ± 0.04 ^D^
21 h	5.84 ± 0.01 ^DE^	5.98 ± 0.05 ^E^	6.10 ± 0.01 ^D^	5.91 ± 0.04 ^C^	6.06 ± 0.01 ^G^	6.14 ± 0.03 ^C^	6.16 ± 0.02 ^D^	6.19 ± 0.04 ^D^
22 h	5.80 ± 0.01 ^DE^	5.98 ± 0.05 ^E^	6.10 ± 0.02 ^D^	5.91 ± 0.04 ^C^	6.06 ± 0.03 ^G^	6.13 ± 0.04 ^C^	6.16 ± 0.01 ^D^	6.17 ± 0.02 ^D^
23 h	5.80 ± 0.01 ^DE^	5.98 ± 0.04 ^E^	6.10 ± 0.02 ^D^	5.90 ± 0.04 ^C^	6.06 ± 0.03 ^G^	6.13 ± 0.04 ^C^	6.16 ± 0.01 ^D^	6.17 ± 0.02 ^D^
24 h	5.77 ± 0.01 ^E^	5.97 ± 0.05 ^E^	6.10 ± 0.02 ^D^	5.93 ± 0.05 ^C^	6.06 ± 0.06 ^G^	6.13 ± 0.04 ^C^	6.17 ± 0.02 ^D^	6.17 ± 0.05 ^D^
*p*-value	<0.001	<0.001	<0.001	<0.001	<0.001	<0.001	<0.001	<0.001

^A–G^ Means followed by distinct letters in the column differ by Tukey’s test (*p* < 0.05). SD: standard deviation.

**Table 3:** Frequency (%) of Botucatu rabbits that presented femorotibiopatelar joint as a function of post-mortem time for each category evaluated. *p*-values are presented.

Time Post-Mortem	Male		Female	
	3 Months	12 Months	3 Months	12 Months
0 h	100 ^A^	100 ^A^	100 ^A^	100 ^A^
1 h	100 ^A^	100 ^A^	100 ^A^	100 ^A^
2 h	100 ^A^	100 ^A^	100 ^A^	100 ^A^
3 h	90 ^A^	90 ^A^	90 ^A^	95 ^A^
4 h	55 ^A^	50 ^A^	65 ^A^	90 ^A^
5 h	35 ^B^	40 ^B^	35 ^B^	56 ^A^
6 h	0 ^B^	0 ^B^	0 ^B^	25 ^B^
7 h	0 ^B^	0 ^B^	0 ^B^	0 ^B^
8 h	0 ^B^	0 ^B^	0 ^B^	0 ^B^
9 h	0 ^B^	0 ^B^	0 ^B^	0 ^B^
10 h	0 ^B^	0 ^B^	0 ^B^	0 ^B^
11 h	0 ^B^	0 ^B^	0 ^B^	0 ^B^
12 h	0 ^B^	0 ^B^	0 ^B^	0 ^B^
13 h	0 ^B^	0 ^B^	3 ^B^	8 ^B^
14 h	0 ^B^	11 ^B^	6 ^B^	8 ^B^
15 h	12 ^B^	13 ^B^	11 ^B^	12 ^B^
16 h	20 ^B^	20 ^B^	15 ^B^	25 ^B^
17 h	30 ^B^	30 ^B^	35 ^B^	40 ^B^
18 h	70 ^A^	65 ^A^	75 ^A^	75 ^A^
19 h	78 ^A^	79 ^A^	89 ^A^	80 ^A^
20 h	88 ^A^	93 ^A^	96 ^A^	94 ^A^
21 h	94 ^A^	99 ^A^	100 ^A^	100 ^A^
22 h	100 ^A^	100 ^A^	100 ^A^	100 ^A^
23 h	100 ^A^	100 ^A^	100 ^A^	100 ^A^
24 h	100 ^A^	100 ^A^	100 ^A^	100 ^A^
*p*-value	<0.001	<0.001	<0.001	<0.001

^A,B^ Frequencies followed by distinct letters in the column differ by Fischer’s Exact test (*p* < 0.05).

## Data Availability

The data that support the findings will be available in Repositorio Institucional da UNESP at http://hdl.handle.net/11449/244719 (accessed on 1 June 2024), following an embargo from the date of publication to allow time for the article to be firstly published.
